# The assessment of pragmatics in Iranian patients with right brain damage

**Published:** 2014-04-03

**Authors:** Davood Sobhani-Rad, Askar Ghorbani, Hassan Ashayeri, Shohereh Jalaei, Behrooz Mahmoodi-Bakhtiari

**Affiliations:** 1 Department of Speech Therapy, School of Rehabilitation, Tehran University of Medical Sciences, Tehran, Iran; 2 Department of Speech Therapy, School of Paramedical Sciences, Mashhad University of Medical Sciences, Mashhad, Iran; 3 Department of Neurology, School of Medicine, Tehran University of Medical Sciences, Tehran, Iran; 4 Department of Rehabilitation, School of Rehabilitation, Iran University of Medical Sciences, Tehran, Iran; 5 Department of Rehabilitation, School of Rehabilitation, Tehran University of Medical Sciences, Tehran, Iran; 6 Department of Performing Arts, School of Fine Arts, Tehran University, Tehran, Iran

**Keywords:** Pragmatics, Psychometric Assessment, Right Brain Damaged Patients

## Abstract

**Background:** Pragmatics is appropriate use of language across a variety of social contexts that provides accurate interpretation of intentions. The occurrence of the right hemisphere lesions can interfere with pragmatic abilities, and particularly with the processing of nonliteral speech acts.

**Methods:** Since the objective of this study was to assess different aspects of pragmatic competence in the right hemisphere damage (RHD) patients, 20 Iranian patients with right hemisphere lesions were examined by adult pragmatic profile (APP) and a novel checklist was introduced for Persian language speaking individuals. Meanwhile, 40 healthy adult individuals, who were age and gender matched with RHD patients, were considered as the control group. After obtaining video records, all subjects were evaluated for 35 pragmatic skills, including 24 verbal, 5 paralinguistic, and 6 nonverbal aspects, by a two-point scale system.

**Results:** Studying RHD patients and their healthy counterparts revealed that the performance by participants with right hemisphere lesions exhibited a high degree of inappropriate pragmatic abilities compared with controls in all domains. Furthermore, RHD patients showed a trend of increasing difficulty in understanding and producing different pragmatic phenomena, including standard communication acts.

**Conclusion:** Present results indicated that the right hemisphere lesions significantly affected pragmatic abilities in verbal, paralinguistic and nonverbal aspects. Such a pattern of performance, which is in line with deficits previously reported for RHD, proved the unquestioned role of the right hemisphere in processing nonliteral language.

## Introduction

Pragmatics is defined as appropriate use of language either to comprehend ideas or to interact in social situations effectively. Pragmatic competence comprises a number of interrelated skills, which manifest in a range of adaptive behaviors, and is considered as the third major component of language ability in addition to phonology, syntax, and semantics.^1^ In language pathology research, there has been increasing interest in recent years in the relationship between cognitive dysfunction and pragmatic impairment, particularly in conditions such as right hemisphere damage (RHD) and traumatic brain injuries.^2^ RHD patients often show normal syntactical and lexical abilities, while they have substantial difficulty managing interactions in their everyday life;^3^ since no report has been published on pragmatic impairments of Iranian patients with RHD, the study aimed to assess communicative abilities, in verbal, paralinguistic and nonverbal aspects, in RHD patients.

Pragmatic abilities are primarily assessed by observational profiles that are based upon discourse samples and assist clinicians to identify the presence and appropriateness of various pragmatic behaviors.^4^ Accordingly, assessment of spontaneous communication allows a wide variety of analyzes that reduces the risk of subjective interpretations or loss of information.^5^ Although several measures of pragmatics have been developed as guidelines for clinical observations, not all language assessment instruments are suitable for comprehensive evaluation of pragmatics, especially when cultural differences are considered. Therefore, to survey pragmatics in Persian language individuals, adult pragmatic profile (APP), that has been recently introduced as a valid and reliable pragmatic assessment tool,^6^ was applied.

## Materials and Methods

***Participants and procedure***

In the present research, all procedures were submitted and approved by the Research Ethics Committee of Tehran University, under protocol number 130/82, and all studies were conducted at the Speech-Language Pathology Clinic of Mashhad University of Medical Sciences (MUMS) and Shariaty Neurology Department of Tehran University. In the current study, 20 RHD patients, who were between 3 and 12 months post-brain injury, were studied for pragmatic abilities. Patient group included 13 males and 7 females aged ranges between 40 and 65 years old (M = 58.2, SD = 6.5). Lesion location was determined by review of magnetic resonance imaging and/or computed tomography scans along with clinical neurological examination. Meanwhile, 40 healthy individuals, including 25 males and 15 females in the same range of age (M = 57.7, SD = 4.9), were considered as the control group. All participants were native Persian language speakers, their education ranged from 5 to 14 years of schooling (M = 9.3, SD = 2.4), provided informed consent and had no history of psychiatric or neurological disease (secondary for RHD patients). Subjects were not gender matched, which is consistent with previous reports that for the communication assessments used, gender was not an influencing variable, unlike age and education.^5^ Speech-language pathologists were trained by authors to gather data and had no previous contact with participants, to avoid the influence of familiarity with the interlocutor.

For pragmatic assessment, a novel adult pragmatic checklist, known as APP, was applied and verbal abilities, such as comprehensibility, contingency, cohesion, redundancy and maintenance, paralinguistic skills, including intelligibility, prosody, pitch and vocal intensity, and nonverbal abilities, such as physical contact, facial expression and gesture, were examined in RHD patients and their healthy counterparts. For psychometric assessment of 35 items in APP, a two-point scale was used and pragmatic modalities were evaluated as appropriate or inappropriate. Instrument used in this study included about 30 min of recording time with each participant, that was repeated 1–2 weeks later, and recordings took place in situations of spontaneous interaction between participants and researchers.

***Statistical analysis***

Data analysis was performed by using SPSS Version 19.0 software. The distribution of collected data was studied by Shapiro-Wilk and Kolmogorov-Smirnov tests, and comparison between RHD patients and control individuals in pragmatic domains was calculated by independent sample t-test.

## Results

The aim of the current attempt was to study the pragmatic impairment in Iranian patients with RHD. To do so, APP, is a novel pragmatic assessment tool suitable for Persian language communities^6^ was used. Pragmatic modalities, in terms of verbal, paralinguistic and nonverbal aspects, were assessed in 20 RHD patients and 40 healthy adult individuals. As soon as recorded language samples were obtained, all participants were studied for 35 communicative items and evaluations were carried out in a two-point scale system. As shown in table 1, most abilities in verbal, paralinguistic and nonverbal subscales were evaluated as inappropriate in RHD patients, quit unlike healthy individuals.

**Figure 1:**
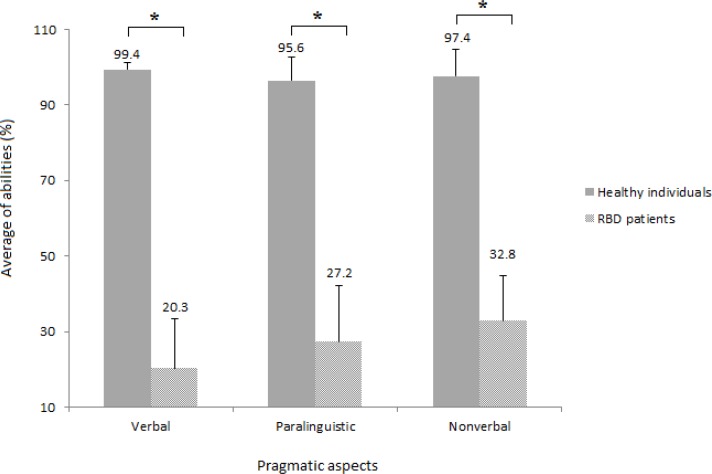
Comparison of pragmatic abilities in verbal, paralinguistic, and nonverbal aspects between right hemisphere damage patients (N = 20) and healthy individuals (N = 40). Data are expressed as mean ± standard deviation. *Indicates significant difference at the 0.01 level (two-tailed).

We have recently reported on the high content and construct validities of APP, and also confirmed its high intra- and inter-reliabilities (Table 2).^6^ Herein, RHD patients were assessed for pragmatic abilities and compared with healthy participants. As presented in figure 1, verbal, paralinguistic and nonverbal abilities were evaluated as 20.3%, 27.2%, and 32.8% in RHD patients, respectively. However, in healthy individuals, verbal, paralinguistic, and nonverbal modalities were reported as 99.4%, 95.6%, and 97.4%, respectively. Comparing communicative behaviors between RHD patients and their healthy counterparts, independent sample t-test revealed significant differences in verbal, paralinguistic and nonverbal aspects.

**Table 1:** APP applied to 20 participants with RHD

**Communicative act patients**	**1**	**2**	**3**	**4**	**5**	**6**	**7**	**8**	**9**	**10**	**11**	**12**	**13**	**14**	**15**	**16**	**17**	**18**	**19**	**20**
**Verbal aspects**																				
1. Variety of speech acts																				
2. Lexical choice																				
3. Introduction																				
4. Maintenance																				
5. Topic shift																				
6. Initiation																				
7. Response																				
8. Termination																				
9. Self-correction																				
10. Pause time																				
11. Interruption																				
12. Request																				
13. Acknowledgment																				
14. Sequencing																				
15. Quantity																				
16. Accuracy																				
17. Cohesion																				
18. Ellipsis																				
19. Tense use																				
20. Turn taking																				
21. Reference																				
22. Stereotypes																				
23. Polite forms																				
24. Sarcasm																				
Percentage	21	8	25	29	100	62	4	4	0	17	50	8	0	4	0	0	0	4	0	46
**Paralinguistic aspects**																				
25. Intelligibility/vocal quality																				
26. Vocal intensity																				
27. Pitch																				
28. Prosody/intonation																				
29. Fluency/rate																				
Percentage	20	100	80	20	0	0	0	0	20	0	80	0	0	0	40	60	0	20	40	40
**Nonverbal aspects**																				
30. Physical contacts																				
31. Physical proximity																				
32. Body posture																				
33. Gestures																				
34. Facial expression																				
35. Eye gaze																				
Percentage	50	0	67	0	50	83	67	0	0	0	0	17	0	17	50	17	17	50	0	67

White: Inappropriate; Black: Appropriate; APP: Adult pragmatic profile; RHD: Right hemisphere damage

**Table 2:** Reliability assessments of APP (Sobhani-Rad et al.)^6^

Construct validity calculated by Spearman's rho Internal reliability calculated by Cronbach's alpha	Verbal-paralinguistic	Verbal-nonverbal	Nonverbal-paralinguistic
	0.47*	0.63*	0.44*
	24 verbal items	5 paralinguistic items	6 nonverbal items
	0.94	0.87	0.86

* Correlation is significant at the 0.01 level (two-tailed). APP: Adult pragmatic profile

## Discussion

Research over the last decade has shown that right hemisphere injuries result in a range of communicative deficits that cannot be adequately explained in terms of linguistic impairment.^7^ Various studies have found that RHD individuals can show impairments such as inappropriate contextual use of language; lack of comprehension of non-literal aspects of language such as metaphor, humor, sarcasm and indirect speech acts; inability to evaluate the plausibility or incongruity of an event in a given context; and inability to make inferences based on a message and, thus, to manage the implicit content of many speech acts.^3^ A number of studies have clearly indicated that RHD individuals may have problems understanding non-literal language, but not literal language, suggesting that only high-level language processing is impaired in RHD. These pragmatic deficits; however, are not present in all RHD subjects and patterns of performance may vary from one individual to another.^2^^,^^8^

This diversity of patterns highlights the importance of studying RHD subjects’ communicative performance on an individual basis. However, pragmatic language problems are very difficult to detect, since language pragmatism is dependent on the specific context and implicit rules; to assess pragmatics, many clinicians have to rely on non-standardized, observational methods that can be challenging for determining service eligibility.^9^^,^^10^ In this regard, proper pragmatic assessment tools, which must be specific to different cultures, can help clinicians and speech pathologists to effectively treat and better study social and cognitive functioning, respectively. As RHD patients’ assorted impairments involves verbal abilities, such as comprehensibility, contingency, cohesion, redundancy and maintenance; paralinguistic skills, including intelligibility, prosody, pitch and vocal intensity; and nonverbal abilities, such as physical contact, facial expression and gesture, in the present work we assessed Iranian RHD patients for pragmatic abilities, while considered adult healthy participants as their control. Our results indicated that RHD patients show inappropriate behavior in verbal, paralinguistic and nonverbal aspects. Such a pattern of performance is in line with deficits found after right-hemisphere lesions in other studies.^8^^,^^11^^-^^14^ For instance, Champagne-Lavau and Joanette, who indicated a close association between pragmatics and executive functions, reported on pragmatic deficit and executive dysfunction in RHD patients.^11^ Furthermore, Tompkins et al. demonstrated that incompatibility interpretation was associated with poorer discourse comprehension performance in patients with RHD.^12^ In another study, Cutica et al. reported on weak performance in extra-linguistic pragmatic abilities in RHD patients in comparison with healthy individuals and left hemisphere damaged patients.^13^

It has been suggested that two-point scales in pragmatic assessments force a decision on an assessor, whereas more scales often lead to an overuse of the intermediate or neutral category, minimizing the likelihood of clear differences emerging.^4^ Moreover, reports indicated that about 30 min of recorded language samples are informative enough to outline the pragmatic profile of individuals by protocols that classify abilities as appropriate or inappropriate,^15^^,^^16^ similar to APP that was used in this study. Although a number of pragmatic tools may have specific psychometric limitations, the fact that APP produced an appropriate index for adult pragmatics was taken as evidence by its high validity and reliability.

Research into the psychometric assessment of pragmatics in RHD patients revealed their impairment in different aspects of communicative performances. However, given the rigor of the methodology presented, authors suggest to replicate this study with larger sample and also with various interlocutors.

## Conclusion

Right hemisphere lesion affects pragmatic abilities, and particularly interferes with the processing of nonliteral speech acts. In the present study, we evaluated verbal, paralinguistic and nonverbal aspects of pragmatic abilities in RHD patients using APP. Our results, which are in agreement with previous reports, revealed that pragmatic competence was significantly affected in RHD patients, as they had difficulty in understanding and producing different communicative phenomena.

## References

[1] Gleason JB, Ratner NB (2012). The Development of Language.

[2] Martin I, McDonald S (2003). Weak coherence, no theory of mind, or executive dysfunction? Solving the puzzle of pragmatic language disorders. Brain Lang.

[3] Monetta L, Champagne-Lavau M (2010). Right hemisphere damage and pragmatics. Pragmatics encyclopedia.

[4] Perkins M (2007). Pragmatic Impairment.

[5] Lopes-Herrera SA, Almeida MA (2008). The use of verbal communicative abilities to increase the mean length of utterance in high-functioning autism and Asperger Syndrome. Pro Fono.

[6] Sobhani-Rad D, Ghorbani A, Ashayeri H, Jalaei S, Mahmoodi-Bakhtiari B, Saifpanahi S (2013). Psychometric assessment of adult pragmatic profile. Journal of American Science.

[7] Griffin R, Friedman O, Ween J, Winner E, Happe F, Brownell H (2006). Theory of mind and the right cerebral hemisphere: refining the scope of impairment. Laterality.

[8] Cote H, Payer M, Giroux F, Joanette Y (2007). Towards a description of clinical communication impairment profiles following right-hemisphere damage. Aphasiology.

[9] Olswang LB, Coggins TE, Timler GR (2014). Outcome Measures for School-Age Children with Social Communication Problems. Topics in Language Disorders.

[10] Young EC, Diehl JJ, Morris D, Hyman SL, Bennetto L (2005). The use of two language tests to identify pragmatic language problems in children with autism spectrum disorders. Lang Speech Hear Serv Sch.

[11] Champagne-Lavau M, Joanette Y (2009). Pragmatics, theory of mind and executive functions after a right-hemisphere lesion: Different patterns of deficits. Journal of Neurolinguistics.

[12] Tompkins CA, Fassbinder W, Lehman BM, Baumgaertner A, Jayaram N (2004). Inference generation during text comprehension by adults with right hemisphere brain damage: activation failure versus multiple activation. J Speech Lang Hear Res.

[13] Cutica I, Bucciarelli M, Bara BG (2006). Neuropragmatics: Extralinguistic pragmatic ability is better preserved in left-hemisphere-damaged patients than in right-hemisphere-damaged patients. Brain Lang.

[14] Champagne M, Nespoulous JL, Joanette Y (2014). Do all right brain-damaged subjects show a deficit in non-literal language comprehension?. Brain and Language.

[15] Meilijson SR, Kasher A, Elizur A (2004). Language performance in chronic schizophrenia: a pragmatic approach. J Speech Lang Hear Res.

[16] McNamara P, Durso R (2003). Pragmatic communication skills in patients with Parkinson's disease. Brain and Language.

